# Presence of Ochratoxin A Residues in Blood Serum of Slaughtered Pigs in Greece

**DOI:** 10.3390/toxins16100421

**Published:** 2024-09-30

**Authors:** Mikela Vlachou, Andreana Pexara, Nikolaos Solomakos, Alexander Govaris, Dimitrios Palaiogiannis, Vassilis Athanasiadis, Stavros I. Lalas

**Affiliations:** 1 Laboratory of Hygiene of Foods of Animal Origin, Faculty of Veterinary Science, University of Thessaly, 43100 Karditsa, Greece; vlachoumikvet@uth.gr (M.V.); nsolom@vet.uth.gr (N.S.); agovaris@vet.uth.gr (A.G.); 2 Department of Food Science and Nutrition, University of Thessaly, Terma N. Temponera Street, 43100 Karditsa, Greece; dipaleog@med.uth.gr (D.P.); vaathanasiadis@uth.gr (V.A.); slalas@uth.gr (S.I.L.)

**Keywords:** ochratoxin A, slaughtered pigs, blood serum, ELISA, HPLC-FD, region

## Abstract

This study aimed to assess the presence of ochratoxin A (OTA) residues in the blood serum of slaughtered pigs in Greece. Samples were obtained from 1695 healthy slaughtered pigs originating from 113 different farms located in 21 geographic regional units in 8 different geographic regions of Greece and were analyzed using an immunosorbent assay (ELISA) and high-performance liquid chromatography with fluorescence detector (HPLC-FD). OTA contamination assessment showed that 782 (46.1%) and 1233 (72.7%) samples were OTA-positive, with a concentration range of 0.20–5.38 μg/L and 0.15–5.96 µg/L according to ELISA and HPLC-FD analysis, respectively. Also, 88 (77.9%) and 108 (95.6%) of farms were found to be OTA-positive by ELISA and HPLC-FD analysis, respectively. The highest OTA serum positivity rate (>98%) and toxin level (5.96 µg/L) determined by HPLC-FD were observed in the Thessaly region, whereas a high prevalence of up to 100% (range 75–100%) was found on farms in the Crete Island region. The detection of OTA in the serum of slaughtered pigs in different regions in Greece poses a risk for animal and human health and highlights the need for constant OTA monitoring in the swine industry and pork meat production facilities.

## 1. Introduction

Ochratoxin A (OTA) is a naturally occurring foodborne mycotoxin found in a wide variety of agricultural commodities and animal feeds in many countries of the world [1,2]. It is produced by several fungal species of the genera *Aspergillus* and *Penicillium*, including *A. ochraceus*, *A. carbonarius*, *A. niger*, *P. verrucosum, and P. nordicum* [3,4]. Important factors for fungal OTA production are temperature, water activity (a_w_), and growth medium composition. *Aspergillus* species predominate in warm climate regions, while *Penicillium* isolates are frequently found in cold climate regions [3].

OTA found in consumed feedstuffs can adversely affect animal health, and pigs are considered the most vulnerable species among food-producing animals. High OTA contamination levels in swine feed components in various countries have been reported [5,6,7,8]. In pigs, OTA may accumulate in tissues due to its high bioavailability, long half-life, and limited conversion rate into the virtually non-toxic OTalpha [8]. OTA residues can be found in slaughtered pigs, mainly in blood, at lower concentrations in the kidneys and liver, and at even lower concentrations in muscle tissue and fat, correlating directly with the dietary contamination level [8,9,10,11]. Consequently, the concentration of OTA in pigs’ blood serum indicates exposure to this mycotoxin; thus, it is considered a useful indicator of ochratoxicosis and its accumulation in the edible organs, especially the kidneys and liver, of slaughtered pigs [8,12,13].

The presence of OTA residues in blood can result in the presence of the toxin in pork meat and derived products, especially those containing pig organs such as the kidneys or liver (“carry-over effect”) [2]. These products are considered important sources of chronic dietary exposure to OTA in humans [1]. OTA presence in food and dietary exposure to OTA pose a potential public health risk since OTA has been classified by the International Agency for Research on Cancer (IARC) [14] as possibly carcinogenic to humans (group 2B). Several studies have also revealed the nephrotoxic, immunotoxic, teratogenic, embryotoxic, and genotoxic nature of OTA [1,15].

The results of studies conducted in several countries revealed various levels of OTA residues in pig edible tissues, and particularly high OTA levels have been determined in blood serum [2,7,16,17]. Several analytical methods have been used for the detection and determination of OTA residues in tissues of pigs. The most widely used analytical methods include high-performance liquid chromatography with fluorescence detection (HPLC-FD), liquid chromatography–mass spectrometry (LC-MS), and enzyme-linked immunosorbent assay (ELISA) [2,9,18]. To our knowledge, in Greece, very few data regarding the presence of OTA in slaughtered pigs are available in the literature. Papatsiros et al. [19] determined 0.54 μg/kg of OTA in the livers of slaughtered pigs from one farm out of eight farms examined using liquid chromatography–triple quadrupole mass spectrometry (LC-MS/MS). In our previous study [20], the presence of OTA in serum samples of slaughtered pigs in Greek regions was verified by ELISA analysis. This study aimed to assess the presence of OTA residues in the blood serum of slaughtered pigs in Greece using ELISA and HPLC-FD.

## 2. Materials and Methods

### 2.1. Sample Collection

Pig serum samples were obtained during November 2018 and April 2021. The samples (n = 1695) were randomly collected from healthy slaughtered pigs originating from 113 swine farms (15 pigs per farm) located in 21 geographic regional units in 8 different geographic regions of Greece (Epirus, Thessaly, Western Macedonia, Central Greece, Central Macedonia, Eastern Macedonia and Thrace, Peloponnese, and Crete). Samples were collected from regions with important swine industries according to data published by the Ministry of Rural Development and Food of Greece in 2017.

Pigs were slaughtered according to official regulations for the veterinary supervision in slaughterhouses and in full line with the provisions of the Council Regulation (EC) No. 1099/2009 on the protection of animals at the time of killing (CEC 2009). According to post-mortem inspection of slaughtered pigs, no signs of macroscopic lesions of the kidneys that would indicate a possible nephropathy or other kidney infection were observed. Samples (50 mL) were collected from the pigs during bleeding in the slaughtering process, and blood clotting took place after 30–60 min at ambient temperature. The separation of serum was made by centrifuging blood (2000× *g* for 5 min). The serum samples were kept at −20 °C until OTA analysis. No preservatives were mixed in. Samples were thawed before OTA analysis.

### 2.2. ELISA Analysis

#### 2.2.1. Sample Preparation

OTA extraction from serum was made by using an ELISA kit [RIDASCREEN^®^ OTA 30/15 (R1311)] combined with an OCHRAPREP^®^ immunoaffinity column (Code: P14/P14), (R-Biopharm, Darmstadt, Germany), as previously described by Vlachou et al. [20]. Briefly, a proper aliquot of serum was initially centrifuged. Then, 1 mL methanol (100%) was mixed with centrifuged serum (0.5 mL) (Scharlab, Barcelona, Spain); the serum sample was further mixed for 30 s and centrifuged (Heraeus Biofuge Stratos High-Speed Centrifuge, Hanau, Germany) for 10 min (3000× *g*/10 °C). The supernatant (0.9 mL) was mixed with 130 mM NaHCO_3_ (10 mL) (Chem-Lab NV, Zedelgem, Belgium).

The solution was flown through the OCHRAPREP^®^ column by using hydrostatic pressure; the column was rinsed with 10 mL 130 mM NaHCO_3_/5% methanol and was subsequently rinsed with 2 mL deionized water. All the column liquid was withdrawn by pressing air. The elution of OTA was achieved with 2 mL methanol (100%) in a new vial. The eluted OΤA sample was dried by using a mild nitrogen flow at 60 °C, and then remained residue was mixed again with 130 mM NaHCO_3_ (0.6 mL).

#### 2.2.2. Determination of OTA

The ELISA analysis for OTA was performed by using the BRIDASCREEN^®^ OTA 30/15 ELISA kit, as described by Vlachou et al. [20]. This kit consists of a micro-titer plate with 96 wells coated with OTA antibodies, as well as a standard water OTA solution (0, 50, 100, 300, 900, and 1800 ng/mL), a peroxidase-conjugated OTA, a substrate/chromogen solution (tetramethylbenzidine), an end solution (1 mol/L), a buffer dilution, and a washing buffer (pH 7.4, 10 mM phosphate buffer). The rest of the chemicals used for the analysis were of an analytical grade. Each sample (50 μL), taken as previously described, was added to each well. The analytical procedure was made following the manufacturer’s instructions and with the use of an auto-analyzer ChemWell 2910 (Awareness Technology, Inc., Palm City, FL, USA). The detection limit (LOD) and serum recovery value provided by the ELISA kit manufacturer were 0.207 ppb and 93%, respectively.

### 2.3. HPLC-FD Analysis

#### 2.3.1. Sample Preparation

For the extraction of OTA for HPLC-FD analysis, the methodology used by Pleadin et al. [13] was applied. All solvents and chemicals used for the preparation of the samples and OTA analysis were of an analytical/HPLC grade.

In brief, serum samples (5 mL) were mixed with 7.5 mL of 1% aqueous NaHCO_3_ (Chem-Lab NV, Zedelgem, Belgium) for 5 min. Then, methanol (17.5 mL) was mixed, vortexed for 1 min, followed by homogenization for 30 min, and centrifugated (10 min, 3500× *g*, room temperature). Then, hexane (10 mL) (Scharlab, Barcelona, Spain) was mixed with the sample. The samples were left after shaking for 3 min. The upper hexane layer was discarded, and the extraction process was repeated. Then, a solution of 0.4 mol/L AgNO_3_ solution (0.25 mL) (Alfa Aesar GmbH & Co KG, Karlsruhe, Germany) was added to the sample (5 mL) followed by centrifugation (10 min, 3500× *g*, room temperature). The supernatant was collected, and purification and concentration were followed using immune-affinity columns (OCHRAPREP^®^, Product Code: P14/P14B, R-Biopharm).

The procedures of purification, elution, and dilution of samples OTA analysis were made following the method used by Pleadin et al. [21]. Sample cleanup was carried out at an average flow rate of 1 drop/s in the following manner: the fluid was drained from a column and supplemented with 1 mL of phosphate-buffered saline (PBS) buffer and 5 mL of the sample solution. The glass bottles used as sample solution containers were washed with 5 mL of PBS (pH 7.4) and applied to the column. The PBS buffer was prepared by solving NaCl (8.0 g), Na_2_HPO_4_ (1.16 g), KH_2_PO_4_ (0.2 g), and KCl (0.2 g) in 1 L of demineralized water (MilliQ system, Millipore, Milford, CT, USA). The column was then washed with 10 mL of PBS buffer and dried for 30 s. OTA was eluted with 1.5 mL of methanol/acetic acid solution (98:2, *v*/*v*) (Chem-Lab NV, Zedelgem, Belgium). The eluted OTA solution was passed in the column three times and then supplemented with 1.5 mL of water. Samples were stored at −18 °C until HPLC-FD analysis.

#### 2.3.2. Determination of OTA

The detection and quantification of OTA by HPLC-FD was performed based on the method developed by Pleadin et al. [13]. The measurement was carried out using a Shimadzu CBM-20A (Shimadzu Europa GmbH, Duisburg, Germany) high-performance liquid chromatography (HPLC) device equipped with an autosampler (SIL-20AC) and a column oven (Shimadzu CTO-20AC). OTA was detected by using a Shimadzu RF-10AXL fluorescence detector (FD) set to 334 nm (excitation) and 460 nm (emission). The chromatographic column used was Phenomenex Luna C18(2) 100 Å, size 250 mm × 4.6 mm with 5 μm particle size (Phenomenex, Inc., Torrance, CA, USA). The applied chromatographic conditions were described by Perši et al. [9].

Separation was carried out under isocratic conditions and the mobile phase at a ratio of acetonitrile (Merck KGaA, Darmstadt, Germany)/water/isopropanol (Fisher Scientific Ltd., Leicestershire, UK)/acetic acid of 46/46/6/2 had a flow rate of 1 mL/min. The column temperature was 40 °C and the sample injection volume was 100 µL.

Standard solutions of OTA (0, 0.05, 0.10, 0.50, and 1.00 μg/L) used for validation purposes and for the determination of OTA were prepared using the standard substance OTA supplied by R-Biopharm (Trilogy^®^ Liquid Standard Ochratoxin A, R-Biopharm Rhône Ltd., Glasgow, UK) by serial 10-fold dilution with methanol.

**Figure 1:**
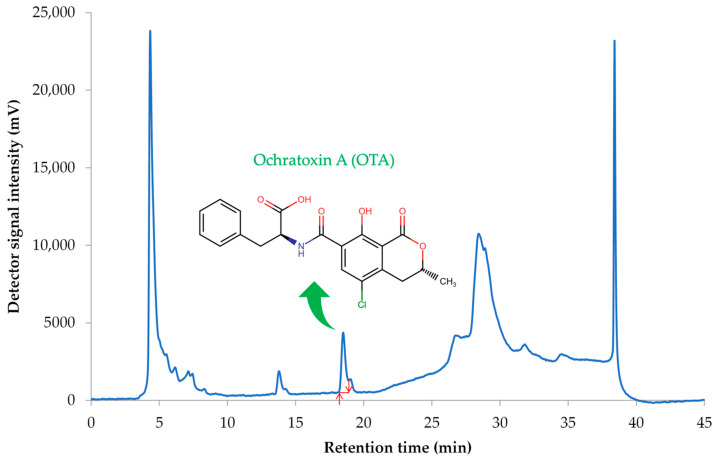
The representative HPLC-FD chromatogram was obtained from a serum sample spiked with OTA at a level of 2.0 μg/L. Red arrows indicate manual integration in chromatographic analysis.

In each analysis, the blank sample (methanol), the standard solutions, and then the analyzed samples were injected. For each fifth injection, a standard solution with an OTA level of 0.10 μg/L was injected to check the stability of the instrument. The retention time of OTA achieved by applying these conditions was 18.4 ± 0.2 min. The limit of quantification (LOQ), the limit of detection (LOD), and the recovery value were 0.15 μg/L, 0.10 μg/L, and 95.6%, respectively [13]. To confirm the reproducibility of our results, in each analysis, the estimation of OTA was carried out in artificially spiked samples at 3 different OTA levels (2.0, 5.0, and 10.0 μg/L) using the standard solution (Figure 1).

### 2.4. Statistical Analysis

Statistical data analysis [mean and median values, coefficient of variation (CV)] determined by HPLC-FD and ELISA and the estimation of the correlation coefficient (R^2^) between the OTA concentrations found by the two methods by means of linear regression was performed using the Statistica Ver 6.1 Software (StatSoft Inc. 1984–2003, Tulsa, OK, USA) as described by Pleadin et al. [13]. Statistical significance was set at 95% (*p* = 0.05).

Values were calculated for positive samples only (>LOD). In the present work, a “positive farm” was characterized when one OTA-contaminated serum sample from at least one animal from the farm was found. Values between LOQ and LOD were replaced with ½ LOQ OTA [22].

## 3. Results

### 3.1. Presence of OTA in the Serum of Slaughtered Pigs

The results on the presence of OTA in the serum of slaughtered pigs in farms estimated by using the ELISA and HPLC-FD methods are presented in Table 1 and Table 2, respectively. OTA contamination assessment by ELISA showed that 782 (46.1%) of the analyzed serum samples (N = 1695) were OTA-contaminated in a range of 0.20–5.38 μg/L (mean value of 0.60 μg/L), and 88 (77.9%) of farms (N = 113) were OTA-positive. According to HPLC-FD analysis, 1233 samples (72.7%) were found to be OTA-positive, with concentrations ranging between 0.15 and 5.96 µg/L and a mean value of 0.51 µg/L. Moreover, 108 (95.6%) farms were found to be OTA-positive when samples were analyzed by HPLC-FD.

**Figure 2:**
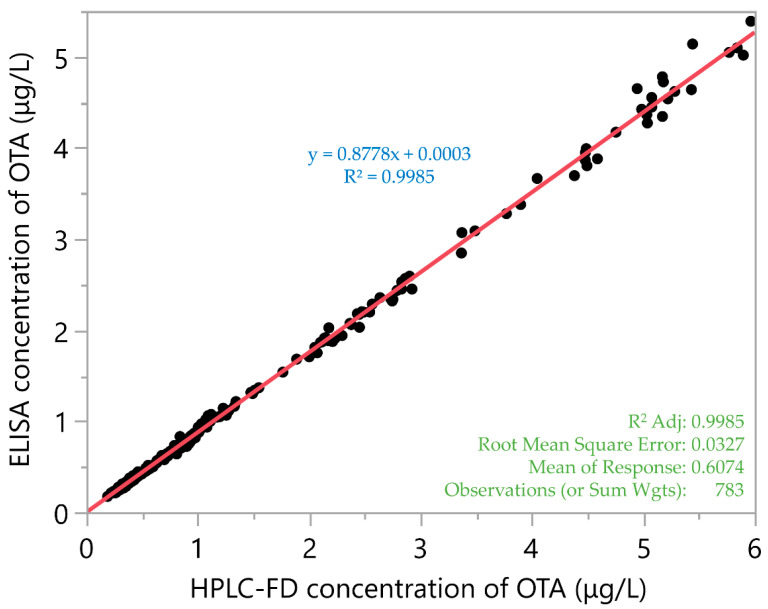
Correlation between OTA concentrations determined in the serum of slaughtered pigs by the ELISA and HPLC-FD methods. Some statistics are also presented.

Despite the lower OTA contents in serum estimated by ELISA as compared to HPLC-FD, a statistically significant correlation (*p* < 0.05) between the concentrations determined by the two methods obtained by means of linear regression (line equation y = 0.8778x + 0.003) was revealed, yielding a high correlation coefficient (R^2^ = 0.9985) (Figure 2).

### 3.2. Regional Distribution of OTA in Serum Samples

ELISA (Table 1) and HPLC-FD analyses (Table 2) revealed variations in positivity rates and OTA contamination levels in serum samples from slaughtered pigs among the different regional units and regions of Greece. The highest positivity rates in serum (75.7%) and farms (100%) determined by ELISA were recorded in the Epirus region, with OTA values ranging between 0.21 and 0.43 µg/L. The region of Thessaly was second highest in the positive range, with 70.9% positive samples and 95.5% positive farms. The lowest serum positivity rate (8.0%) and the lowest concentration levels (0.21–0.25 µg/L) were found in samples from the Eastern Macedonia and Thrace regions. In the serum samples of slaughtered pigs analyzed by HPLC-FD, the highest positivity rates for OTA were observed in the regions of Thessaly and Crete, both exceeding 98%. OTA concentrations ranged from 0.15 to 5.96 µg/L in Thessaly and from 0.15 to 1.11 µg/L in Crete. In the region of Thessaly, the highest toxin level (5.96 µg/L) was detected by HPLC-FD, along with the highest CV (1.33). The lowest positivity rate was observed in samples from Central Greece (39.3%), where OTA concentrations ranged from 0.15 to 0.37 µg/L.

### 3.3. Prevalence of OTA Contamination in Serum Samples on Farms by Region

The prevalence of OTA contamination in the serum of slaughtered pigs on farms, i.e., the number of OTA-positive serum samples out of 15 total samples examined from each farm, was evaluated. A very different regional distribution of prevalence (Table 3) was observed. In the Thessaly region, out of 22 farms included in our study, the highest percentage (range 75–100%) of tested serum samples was found to be OTA-positive: this result was reached on 12 (54.6%) and 21 (95.5%) farms tested using ELISA and HPLC-FD, respectively. A high prevalence (range 75–100%) on farms, of up to 100%, was determined by HPLC-FD in the region of Crete. In the Eastern Macedonia and Thrace region, a low prevalence (range 0–25%) was recorded in 4 out of 5 farms, as determined by ELISA.

## 4. Discussion

The mean OTA levels in pig serum estimated either by ELISA (0.60 μg/L) or HPLC-FD (0.51 μg/L) analyses were low. It is important to note that after 42 days of feeding pigs with feed supplemented with 400 and 800 μg/kg OTA, OTA levels reached 1.21 and 2.04 μg/mL, respectively [23]. Feeding with OTA-contaminated feed (250 μg OTA/kg of feed) for 30 days of pig fattening resulted in a mean OTA concentration in serum of 4.77 ± 1.57 μg/L, estimated by using HPLC-FD analysis [13]. After oral OTA administration (0.78 mg OTA per pig) for 22 days, the mean OTA concentration in serum was 0.875 ± 0.293 μg/L, as estimated with ELISA analysis [24]. Official limits of OTA levels in meat have not been set by international food safety authorities [25]. In European Union countries, Commission Regulation 2022/1370 amending previous regulation 2006/1881 sets maximum OTA limits in various food commodities, but no OTA limits have been established for meat. However, certain countries have set OTA limits of 5 μg/kg (Romania) and 10 μg/kg (Denmark) in pig kidneys, 10 μg/kg (Estonia) and 5 μg/kg (Romania) in pig livers, and 5 μg/kg (Romania, Slovakia) and 1 μg/kg (Italy) in pig meat [26].

Krüger et al. [27] examined 87 blood serum samples from farm pigs in Rio de Janeiro, Brazil, and found OTA in 4 samples (4.6%), with varying concentrations ranging from 0.1546 to 1.4851 ng/mL. Among positive samples, the mean was 0.5739 ng/mL, with a standard deviation of 0.6130, while the remaining samples presented levels below the LOQ. Grajewski et al. [28] used HPLC-FD to examine OTA contamination levels in the serum of wild boars hunted in five northwest regions of Poland during November and December 2006 (N = 39) and throughout 2007 (N = 62). The mean OTA levels in serum were 6.15 ng/mL and 5.91 ng/mL in 2006 and 2007, respectively, while the highest OTA record in a serum sample was 1.170 ng/mL. They also reported that OTA levels in the serum of wild boars were 3 times higher than the OTA level found in the serum of farm pigs in Poland. Milićević et al. [29] examined OTA presence in the serum of pigs (n = 90) slaughtered in Serbia using HPLC. OTA levels in serum were 31%, while the maximum OTA levels were 220.8 ng/mL. OTA presence in the serum of pigs in Piedmont, in northwest Italy (on 4 organic and 11 conventional farms) from September 2006 to March 2009 was examined by Pozzo et al. [30]. OTA was found in all pig serum samples at levels ranging from 0.03 to 0.87 ng/mL and from 0.15 to 6.24 ng/mL in conventional and organic farms, respectively.

In the present study, the positivity rate of OTA presence in farms determined by HPLC-FD was high (95.6%). In an earlier study, only 47 out of 279 (16.8%) Swedish pig farms were found to be OTA-positive in blood in amounts greater than or equal to 2 ng/mL, as identified by a spectrofluorometric procedure [31]. OTA was found in all pig serum samples at levels ranging from 0.03 to 0.87 ng/mL and from 0.15 to 6.24 ng/mL in conventional and organic farms, respectively. In Romania, 98% [16] and 94% [17] of pig serum samples were found to be OTA-positive, as analyzed by HPLC-FLD. In a study by Curtui et al. [16], OΤA content ranged from 0.05 to 13.4 μg/L, whereas 85% of positive samples contained OTA under 5 μg/L. Curtui and Gareis (2001) [17] reported OTA levels in the range of 0.1–13.4 μg/L. In Canada, in an earlier study, pig serum analysis revealed that 3.6–65% of samples were OTA-positive, with concentrations between 5.4 and 20 μg/L [32]. A lower incidence of OTA (31.1%) compared to the findings of the present study was found in pig serum in Serbia, but contamination levels were higher and ranged between 0.22 and 220.8 μg/L [33].

The presence of OTA in pig blood or tissues is directly related to the exposure of pigs to OTA-contaminated feed [8]. The mean OTA concentration differences in the serum of slaughtered pigs as determined by both methods are ascribed to different factors such as OTA concentration in the diet, the length of exposure, and exposure during slaughter [18,33,34,35]. Pigs’ dietary exposure to OTA may be associated with OTA toxigenic mold growth on feed grains due to environmental and climate conditions (e.g., excessive rainfall during grain harvesting), the status of grain drying and humidity levels during feed storage, and OTA-contaminated ingredients in feed [33,35].

Various studies have reported different OTA levels in swine feed across multiple countries [5,6,7,8,30,36,37], with higher OTA concentrations detected in organic feed samples compared to conventional feed samples [30,38,39].

The different OTA levels found in pig serum may be also due to the analytical methods used [2,26]. In the present study, the higher OTA positivity rates in samples determined by HPLC-FD compared to those determined by ELISA may be explained by the lower HPLC-FD LOD (0.10 μg/L HPLC-FD versus 0.20 μg/L ELISA). Also, the lower OTA contents determined by ELISA are consistent with results from previous studies [9,13,18,40] indicating that ELISA tends to underestimate OTA content in body fluids and tissues compared to HPLC-FD. However, the OTA concentrations in pig serum obtained using both analytical methods were highly correlated. These results are consistent with previous reports [9,13], reinforcing the finding that both applied methods are suitable for the determination of OTA in pig serum: ELISA as a tool for screening purposes and HPLC-FD as the confirmatory method [1,9,18,20].

Overall, our results reinforce the previously suggested view that OTA determination in serum could serve as a rapid tool for OTA monitoring in slaughtered pigs, as well as in pig farms, using ELISA as a screening tool and HPLC-FD as a confirmatory method [9,13].

The results of the present study showed a different regional distribution of OTA presence in the serum of slaughtered pigs in Greece. Similarly, geographical variations in OTA occurrence in slaughtered pigs’ serum were also reported in Romania, Serbia, and Brazil [12,16,33], but, in contrast, no regional difference was reported in Sweden in the early study of Hult et al. [31]. In agreement with our results, Polovinski Horvatovic et al. [35] found that the prevalence of OTA contamination in pig kidneys varied from farm to farm in different regions of Serbia.

Since weather conditions can affect the growth of toxigenic OTA fungi and OTA production in feeds during storage, different regional weather conditions may explain the differences found between OTA contamination levels in pig serum from different regions [20]. These regional differences may also be associated with differences in OTA-contaminated feed formulas [30,35]. The differences in positivity rates and OTA contamination levels found among samples originating from farms in the same regional unit can also be associated with the feed management on the farms [35]. Moreover, the sampling time may have affected the regional distribution of OTA in serum samples, since temporal and seasonal variation in OTA content in animal tissues has been reported by other researchers [7,34].

To the best of our knowledge, this study was the first assessment of the presence of OTA in the serum of slaughtered pigs in Greece using two methods (ELISA and HPLC-FD). The detection of OTA in the serum of slaughtered pigs in different regions in Greece poses a risk for animal and human health and highlights the need for constant OTA monitoring in the production of pork meat. Further research should be focused on preventive measures to avoid OTA contamination in feed.

## Figures and Tables

**Table 1:** Presence of OTA in serum of pigs slaughtered in Greece as determined by ELISA.

REGION	REGIONAL UNIT	FARMS		SAMPLES						
		Ν	n (%)	N	n (%)	Mean * (μg/L)	SD *	Median * (μg/L)	CV *	Range * (μg/L)
**EPIRUS**	Arta	3	3 (100)	45	44 (97.8)	0.30	0.04	0.28	0.14	0.22–0.41
	Ioannina	5	5 (100)	75	42 (56.0)	0.27	0.04	0.26	0.16	0.20–0.40
	Preveza	9	9 (100)	135	107 (79.3)	0.29	0.04	0.28	0.15	0.21–0.43
	Total	17	17 (100)	255	193 (75.7)	0.29	0.04	0.28	0.15	0.21–0.43
**THESSALY**	Karditsa	7	7 (100)	105	105 (100)	2.26	1.40	1.93	0.61	0.58–5.38
	Larissa	7	7 (100)	105	53 (50.5)	0.32	0.11	0.27	0.35	0.21–0.73
	Trikala	7	7 (100)	105	70 (66.7)	0.34	0.13	0.28	0.37	0.21–0.64
	Magnesia	1	1 (100)	15	6 (40.0)	0.23	0.01	0.23	0.04	0.22–0.24
	Total	22	21 (95.5)	330	234 (70.9)	1.20	1.34	0.53	1.11	0.21–5.38
**CENTRAL GREECE**	Euboea	15	5 (33.3)	225	29 (12.9)	0.26	0.03	0.25	0.13	0.22–0.33
	Phthiotis	4	4 (100)	60	6 (10.0)	0.22	0.01	0.22	0.08	0.20–0.25
	Total	19	9 (47.4)	285	35 (12.3)	0.25	0.03	0.25	0.13	0.20–0.33
**CENTRAL MACEDONIA**	Thessaloniki	7	7 (100)	105	64 (61.0)	0.31	0.06	0.30	0.20	0.21–0.48
	Pieria	11	6 (54.5)	165	24 (14.5)	0.29	0.10	0.27	0.34	0.21–0.7
	Serres	6	5 (83.33)	90	29 (32.2)	0.34	0.19	0.25	0.56	0.20–0.89
	Chalkidiki	2	0 (0.0)	30	0 (0.0)	-	-	-	-	-
	Total	26	18 (69.2)	390	117 (30.0)	0.31	0.11	0.29	0.37	0.20–0.89
**WESTERN GREECE**	Aetolia-Acarnania	11	8 (72.7)	165	104 (63.0)	0.54	0.26	0.58	0.48	0.21–1.07
	Elis	4	4 (100)	60	17 (28.3)	0.28	0.06	0.25	0.22	0.21–0.40
	Total	15	12 (80.0)	225	121 (53.8)	0.50	0.26	0.43	0.51	0.21–1.07
**EASTERN MACEDONIA AND THRACE**	Drama	3	1 (33.3)	45	1 (2.2)	-	-	-	-	0.24
	Xanthi	2	2 (100)	30	5 (16.7)	0.23	0.01	0.23	0.06	0.21–0.25
	Total	5	3 (60.0)	75	6 (8.0)	0.23	0.01	0.23	0.05	0.21–0.25
**PELOPONNESE**	Corinthia	2	2 (100)	30	29 (96.7)	0.30	0.05	0.28	0.18	0.22–0.43
	Laconia	3	2 (66.7)	45	8 (17.8)	0.25	0.04	0.24	0.16	0.22–0.33
	Total	5	4 (80.0)	75	37 (49.3)	0.29	0.05	0.27	0.19	0.22–0.43
**CRETE**	Heraklion	4	4 (100)	60	39 (65.0)	0.45	0.28	0.27	0.62	0.20–0.99
	Total	4	4 (100)	60	39 (65.0)	0.45	0.28	0.27	0.62	0.20–0.99
**WHOLE TOTAL**		113	88 (77.9)	1695	782 (46.1)	0.60	0.84	0.30	1.39	0.20–5.38

* Calculated for positive samples only. CV = coefficient of variation; N = total number of analyzed samples; n = number of positive samples (see the Section 2).

**Table 2:** Presence of OTA in serum of pigs slaughtered in Greece as determined by HPLC-FD.

Region	Regional Unit	Farms		Samples						
		Ν	n (%)	N	n (%)	Mean * (μg/L)	SD *	Median * (μg/L)	CV *	Range * (μg/L)
Epirus	Arta	3	3 (100)	45	45 (100)	0.33	0.05	0.32	0.15	0.19–0.44
	Ioannina	5	5 (100)	75	65 (88.0)	0.26	0.07	0.27	0.25	0.15–0.45
	Preveza	9	9 (100)	135	135 (100)	0.30	0.07	0.30	0.22	0.17–0.50
	Total	17	17 (100)	255	246 (96.5)	0.30	0.07	0.30	0.23	0.15–0.50
Thessaly	Karditsa	7	7 (100)	105	105 (100)	2.58	1.58	2.23	0.61	0.65–5.96
	Larissa	7	7 (100)	105	99 (94.3)	0.29	0.13	0.26	0.44	0.15–0.84
	Trikala	7	7 (100)	105	105 (100)	0.33	0.15	0.27	0.45	0.16–0.72
	Magnesia	1	1 (100)	15	15 (100)	0.24	0.03	0.23	0.13	0.18–0.29
	Total	22	22 (100)	330	324 (98.2)	1.04	1.40	0.33	1.33	0.15–5.96
Central Greece	Euboea	15	11 (73.3)	225	70 (31.1)	0.23	0.06	0.21	0.26	0.15–0.37
	Phthiotis	4	4 (100)	60	42 (70.0)	0.19	0.03	0.18	0.19	0.15–0.29
	Total	19	15 (78.9)	285	112 (39.3)	0.22	0.05	0.19	0.25	0.15–0.37
Central Macedonia	Thessaloniki	7	7 (100)	105	88 (83.8)	0.31	0.09	0.29	0.30	0.15–0.53
	Pieria	11	11 (100)	165	74 (44.8)	0.23	0.09	0.19	0.38	0.15–0.78
	Serres	6	6 (100)	90	73 (81.1)	0.28	0.18	0.22	0.67	0.15–1.01
	Chalkidiki	2	1 (50.0)	30	3 (10.0)	0.79	1.04	0.19	1.32	0.17–2.00
	Total	26	25 (96.2)	390	238 (61.0)	0.28	0.17	0.23	0.61	0.15–2.00
Western Greece	Aetolia-Acarnania	11	11 (100)	165	132 (80.0)	0.53	0.31	0.38	0.58	0.15–1.25
	Elis	4	4 (100)	60	37 (61.7)	0.25	0.07	0.23	0.29	0.15–0.45
	Total	15	15 (100)	225	169 (75.1)	0.47	0.30	0.31	0.64	0.15–1.25
Eastern Macedonia and Thrace	Drama	3	3 (100)	45	12 (26.7)	0.19	0.03	0.18	0.18	0.15–0.27
	Xanthi	2	2 (100)	30	20 (66.7)	0.20	0.03	0.19	0.18	0.15–0.28
	Total	5	5 (100)	75	32 (42.7)	0.20	0.03	0.18	0.18	0.15–0.28
Peloponnese	Corinthia	2	2 (100)	30	30 (100)	0.33	0.06	0.33	0.19	0.19–0.47
	Laconia	3	3 (100)	45	23 (51.1)	0.22	0.06	0.19	0.27	0.15–0.37
	Total	5	5 (100)	75	53 (70.7)	0.28	0.08	0.28	0.28	0.15–0.47
Crete	Heraklion	4	4 (100)	60	59 (98.3)	0.41	0.30	0.25	0.73	0.15–1.11
	Total	4	4 (100)	60	59 (98.3)	0.41	0.30	0.25	0.73	0.15–1.11
Whole Total		113	108 (95.6)	1695	1233 (72.7)	0.51	0.8	0.28	1.57	0.15–5.96

* Calculated for positive samples only. CV = coefficient of variation; N = total number of analyzed samples; n = number of positive samples (see the “Material and Methods” section).

**Table 3:** Prevalence of OTA contamination in serum of slaughtered pigs on farms by region in Greece.

Region	Prevalence Rate (Range)							
	ELISA				HPLC-FD			
	0–25%	25–50%	50–75%	75–100%	0–25%	25–50%	50–75%	75–100%
	n (%)							
Epirus (N = 17)	1 (5.9)	0 (0.0)	8 (47.1)	8 (47.1)	0 (0.0)	0 (0.0)	1 (5.9)	16 (94.1)
Thessaly (N = 22)	2 (9.0)	5 (22.7)	3 (13.6)	12 (54.6)	0 (0.0)	0 (0.0)	1 (4.6)	21 (95.5)
Central Greece (N = 19)	15 (79.0)	3 (16.0)	1 (5.3)	0 (0.0)	7 (36.6)	4 (21.1)	4 (21.1)	4 (21.1)
Central Macedonia (N = 26)	13 (50.0)	5 (19.2)	5 (19.2)	3 (11.5)	4 (15.4)	6 (23.1)	4 (15.4)	12 (46.2)
Western Greece (Ν = 15)	5 (33.3)	2 (13.3)	2 (13.3)	6 (40.0)	1 (6.7)	4 (26.7)	0 (0.0)	10 (66.7)
Eastern Macedonia and Thrace (Ν = 5)	4 (80.0)	1 (20.0)	0 (0.0)	0 (0.0)	2 (40.0)	1 (20.0)	2 (40.0)	0 (0.0)
Peloponnese (Ν = 5)	2 (40.0)	1 (20.0)	0 (0.0)	2 (40.0)	1 (20.0)	0 (0.0)	2 (40.0)	2 (40.0)
Crete (Ν = 4)	0 (0.0)	1 (25.0)	2 (50.0)	1 (25.0)	0 (0.0)	0 (0.0)	0 (0.0)	4 (100)
Total (Ν = 113)	42 (37.2)	18 (15.9)	21 (18.6)	32 (28.3)	15 (13.3)	15 (13.3)	14 (12.4)	69 (61.1)

N = total number of tested farms; n = number of farms.

## Data Availability

The data presented in this study are available on request from the corresponding author due to our university requirements.

## References

[1] Schrenk D., Bodin L., Chipman J.K., del Mazo J., Grasl-Kraupp B., Hogstrand C., Hoogenboom L.R., Leblanc J.C., Nebbia C.S., EFSA Panel on Contaminants in the Food Chain (CONTAM) (2020). Risk assessment of aflatoxins in food. EFSA J..

[2] Vlachou M., Pexara A., Solomakos N., Govaris A. (2022). Ochratoxin A in Slaughtered Pigs and Pork Products. Toxins.

[3] Wang Y., Wang L., Liu F., Wang Q., Selvaraj J.N., Xing F., Zhao Y., Liu Y. (2016). Ochratoxin A Producing Fungi, Biosynthetic Pathway and Regulatory Mechanisms. Toxins.

[4] Atumo S. (2020). A Review of Ochratoxin A Occurrence, Condition for the Formation and Analytical Methods. Int. J. Agric. Sci. Food Technol..

[5] Li X., Zhao L., Fan Y., Jia Y., Sun L., Ma S., Ji C., Ma Q., Zhang J. (2014). Occurrence of mycotoxins in feed ingredients and complete feeds obtained from the Beijing region of China. J. Anim. Sci. Biotechnol..

[6] Leiva A., Méndez G., Rodríguez C., Molina A., Granados-Chinhilla F. (2019). Chemical assessment of mycotoxin contaminants and veterinary residues in Costa Rican animal feed. Food Contam..

[7] Ganesan A.R., Balasubramanian B., Park S., Jha R., Andretta I., Bakare A.G., Kim I.H. (2021). Ochratoxin A: Carryover from animal feed into livestock and the mitigation strategies. Anim. Nutr..

[8] Schrenk D., Bignami M., Bodin L., Chipman J.K., del Mazo J., Grasl-Kraupp B., Hogstrand C., Hoogenboom L.R., Leblanc J.-C., EFSA Panel on Contaminants in the Food Chain (CONTAM) (2023). Risks for animal health related to the presence of ochratoxin A (OTA) in feed. EFSA J..

[9] Perši N., Pleadin J., Kovačević D., Scortichini G., Milone S. (2014). Ochratoxin A in raw materials and cooked meat products made from OTA-treated pigs. Meat Sci..

[10] Altafini A., Armorini S., Zaghini A., Sardi L., Roncada P. (2017). Tissue distribution of ochratoxin A in pigs after administration of two-levels contaminated diets. World Mycotoxin J..

[11] Hort V., Nicolas M., Minvielle B., Maleix C., Desbourdes C., Hommet F., Dragacci S., Dervilly-Pinel G., Engel E., Guérin T. (2018). Ochratoxin A determination in swine muscle and liver from French conventional or organic farming production systems. J. Chromatogr. B.

[12] Krüger C.D., Cavaglieri L.R., Direito G.M., Keller K.M., Dalcero A.M., da Rocha Rosa C.A. (2010). Ochratoxin A in serum of swine from different Brazilian states. J. Vet. Diagn. Investig..

[13] Pleadin J., Kudumija N., Kovacevic D., Scortichini G., Milone S., Kmetic I. (2016). Comparison of ochratoxin A levels in edible pig tissues and in biological fluids after exposure to a contaminated diet. Mycotoxin Res..

[14] IARC (International Agency for Research on Cancer) Agents Classified by the IARC Monographs, Volumes 1–136. https://monographs.iarc.who.int/list-of-classifications.

[15] Heussner A.H., Bingle L.E. (2015). Comparative Ochratoxin Toxicity: A Review of the Available Data. Toxins.

[16] Curtui V.G., Gareis M., Usleber E., Märtlbauer E. (2001). Survey of Romanian slaughtered pigs for the occurrence of mycotoxins ochratoxins A and B, and zearalenone. Food Addit. Contam..

[17] Curtui V.G., Gareis M. (2001). A simple HPLC method for the determination of the mycotoxins ochratoxin A and B in blood serum of swine. Food Addit. Contam..

[18] Matrella R., Monaci L., Milillo M.A., Palmisano F., Tantillo M.G. (2006). Ochratoxin A determination in paired kidneys and muscle samples from swines slaughtered in southern Italy. Food Control.

[19] Papatsiros V.G., Stylianaki I., Tsekouras N., Papakonstantinou G., Gómez-Nicolau N.S., Letsios M., Papaioannou N. (2021). Exposure Biomarkers and Histopathological Analysis in Pig Liver after Exposure to Mycotoxins Under Field Conditions: Special Report on Fumonisin B1. Foodborne Pathog. Dis..

[20] Vlachou M., Pexara A., Solomakos N., Govaris A. (2023). Occurrence and Contamination Level of Ochratoxin A in Tissues of Slaughtered Pigs in Greece. Acta Vet. Eurasia.

[21] Pleadin J., Perši N., Kovačević D., Vahčić N., Scortichini G., Milone S. (2013). Ochratoxin A in traditional dry-cured meat products produced from sub-chronic-exposed pigs. Food Addit. Contam. Part A.

[22] Warensjö Lemming E., Montano Montes A., Schmidt J., Cramer B., Humpf H.U., Moraeus L., Olsen M. (2020). Mycotoxins in blood and urine of Swedish adolescents-possible associations to food intake and other background characteristics. Mycotoxin Res..

[23] Gan F., Hou L., Zhou Y., Liu Y., Huang D., Chen X., Huang K. (2017). Effects of ochratoxin A on ER stress, MAPK signaling pathway and autophagy of kidney and spleen in pigs. Environ. Toxicol..

[24] Perši N., Pleadin J., Vulić A., Kmetič I., Šimić B. (2012). Determination of ochratoxin A in serum and urine of pigs. World Mycotox J..

[25] Delfino D., Lucchetti D., Mauti T., Manusco M., Di Giustino P., Vaccari S., Bonnani R.C., Neri B., Russo K. (2022). Investigation of ochratoxin A in commercial cheeses and pork meat products by liquid chromatography–tandem mass spectrometry. J. Food Sci..

[26] Agriopoulou S., Stamatelopoulou E., Varzakas T. (2020). Advances in Occurrence, Importance, and Mycotoxin Control Strategies: Prevention and Detoxification in Foods. Foods.

[27] Krüger C.D., Sobreiro L.G., Tortelly R., Fernandes A.M., Da Rocha Rosa C.A. (2015). Níveis séricos de ocratoxina A e lesões em suínos no Rio de Janeiro, Brasil. [Serum levels of ochratoxin A and lesion in swine in Rio de Janeiro, Brazil]. Rev. Bras. Med. Vet..

[28] Grajewski J., Twaruzek M., Kosicki R. (2012). High levels of ochratoxin A in blood serum and kidneys of wild boars Sus scrofa in Poland. Wildl. Biol..

[29] Milićević D.R., Stefanović S., Janković S., Radičević T. (2012). Risk analysis and exposure assessment of ochratoxin A in Serbia. Vet. World.

[30] Pozzo L., Cavallarin L., Nucera D., Antoniazzi S., Schiavone A. (2010). A survey of ochratoxin A contamination in feeds and sera from organic and standard swine farms in northwest Italy. J. Sci. Food Agric..

[31] Hult K., Hökby E., Gatenbeck S., Rutqvist L. (1980). Ochratoxin A in blood from slaughter pigs in Sweden: Use in evaluation of toxin content of consumed feed. Appl. Environ. Microbiol..

[32] Frohlich A.A., Marquardt R.R., Ominski K.H. (1991). Ochratoxin A as a contaminant in the human food chain: A Canadian perspective. IARC Sci. Publ..

[33] Milićević D., Jurić V., Stefanović S., Jovanović M., Janković S. (2008). Survey of slaughtered pigs for occurrence of ochratoxin A and porcine nephropathy in Serbia. Int. J. Mol. Sci..

[34] Pfohl-Leszkowicz A., Manderville R.A. (2007). Ochratoxin A: An overview on toxicity and carcinogenicity in animals and humans. Mol. Nutr. Food Res..

[35] Polovinski-Horvatovic M., Radovic I., Glamocic D., Jajic I., Krstovic S., Mirkov M., Vasiljevic V. The occurrence of ochratoxin A in kidneys of healthy pigs from Vojvodina province, Serbia. IOP Conference Series: Earth and Environmental Science, Proceedings of the 60th International Meat Industry Conference MEATCON2019, Kopaonik, Serbia, 22–25 September 2019.

[36] Rosa C.A., Keller K.M., Keller L.A., González Pereyra M.L., Pereyra C.M., Dalcero A.M., Cavaglieri L.R., Lopes C.W. (2009). Mycological survey and ochratoxin A natural contamination of swine feedstuffs in Rio de Janeiro State, Brazil. Toxicon.

[37] Eskola M., Kos G., Elliott C.T., Hajšlová J., Mayar S., Krska R. (2020). Worldwide contamination of food-crops with mycotoxins: Validity of the widely cited ‘FAO estimate’ of 25. Crit. Rev. Food Sci. Nutr..

[38] Jørgensen K., Jacobsen J.S. (2002). Occurrence of ochratoxin A in Danish wheat and rye, 1992–1999. Food Addit. Contam..

[39] Czerwiecki L., Czajkowska D., Witkowska-Gwiazdowska A. (2002). On ochratoxin A and fungal flora in Polish cereals from conventional and ecological farms. Food Addit. Contam..

[40] Szőke Z., Babarczi B., Mézes M., Lakatos I., Poór M., Fliszár-Nyúl E., Oldal M., Czéh Á., Bodó K., Nagyéri G. (2022). Analysis and Comparison of Rapid Methods for the Determination of Ochratoxin a Levels in Organs and Body Fluids Obtained from Exposed Mice. Toxins.

